# Adsorption of Brilliant Green Dye onto a Mercerized Biosorbent: Kinetic, Thermodynamic, and Molecular Docking Studies

**DOI:** 10.3390/molecules28104129

**Published:** 2023-05-16

**Authors:** Andra-Cristina Enache, Corneliu Cojocaru, Petrisor Samoila, Victor Ciornea, Roxana Apolzan, Georgeta Predeanu, Valeria Harabagiu

**Affiliations:** 1 Laboratory of Inorganic Polymers, “Petru Poni” Institute of Macromolecular Chemistry, 41A Grigore Ghica Voda Alley, 700487 Iasi, Romania; humelnicu.andra@icmpp.ro (A.-C.E.); hvaleria@icmpp.ro (V.H.); 2 Faculty of Biology and Chemistry, “Ion Creanga” State Pedagogical University, 1 Ion Creangă Street, MD-2069 Chisinau, Moldova; 3 SC Cosfel Actual SRL, 95-97 Grivitei Street, 010705 Bucharest, Romania; 4 Research Center for Environmental Protection and Eco-Friendly Technologies (CPMTE), University Politehnica of Bucharest, 1 Polizu Street, 011061 Bucharest, Romania; gpredeanu@gmail.com

**Keywords:** pistachio shells, biosorbent, brilliant green, optimization, molecular docking

## Abstract

This study reports the valorization of pistachio shell agricultural waste, aiming to develop an eco-friendly and cost-effective biosorbent for cationic brilliant green (BG) dye adsorption from aqueous media. Pistachio shells were mercerized in an alkaline environment, resulting in the treated adsorbent (PS_NaOH_). The morphological and structural features of the adsorbent were analyzed using scanning electron microscopy, Fourier transform infrared spectroscopy, and polarized light microscopy. The pseudo-first-order (PFO) kinetic model best described the adsorption kinetics of the BG cationic dye onto PS_NaOH_ biosorbents. In turn, the equilibrium data were best fitted to the Sips isotherm model. The maximum adsorption capacity decreased with temperature (from 52.42 mg/g at 300 K to 46.42 mg/g at 330 K). The isotherm parameters indicated improved affinity between the biosorbent surface and BG molecules at lower temperatures (300 K). The thermodynamic parameters estimated on the basis of the two approaches indicated a spontaneous (Δ*G* < 0) and exothermic (Δ*H* < 0) adsorption process. The design of experiments (DoE) and the response surface methodology (RSM) were employed to establish optimal conditions (sorbent dose (*SD*) = 4.0 g/L and initial concentration (*C*_0_) = 10.1 mg/L), yielding removal efficiency of 98.78%. Molecular docking simulations were performed to disclose the intermolecular interactions between the BG dye and lignocellulose-based adsorbent.

## 1. Introduction

The consequences of urbanization, rapid population growth, and the concomitant need for economic development have led to the significant pollution of natural waters. Therefore, the development of sustainable methods for water and wastewater treatment is imperative nowadays [1]. Along with the progress of the industrial sector, most natural-origin dyes have been supplanted by synthetic dyes as a result of rising demands and prohibitive costs associated with extracting natural compounds [2]. Therefore, synthetic dyes are considered a worrisome polluting factor in wastewater, as they are widely used in various industries (e.g., textiles, pharmaceuticals, cosmetics, paper, plastic, paint, and leather) [2,3,4]. It is estimated that the textile industry alone utilizes more than 10^7^ kg of dye per year worldwide, of which 10^6^ kg is discharged into water streams [5]. As a consequence, synthetic dyes from wastewater have led to an increase in waterborne diseases worldwide, and implicitly in morbidity and mortality [6,7,8].

Cationic dyes are recognized to be more harmful than anionic dyes, possessing high tinctorial values (1 mg/L) [9]. Among these is brilliant green (BG), a triphenylmethane dye with widespread usage in human and veterinary medicine when used in low-concentration solutions (e.g., antiseptic, antifungal, and anthelmintic properties) [10,11]. Additionally, BG is extensively used as a coloring agent for textile materials and paper (approximately 1 kg/ton of paper), as well as in the rubber and plastic industries [12,13,14]. However, this dye is classified as highly toxic, causing harmful effects in humans when in contact with the skin and eyes, by inhalation, or by ingestion (the probable lethal dose is 50–500 mg/kg) [15,16]. It has been demonstrated that the toxic effect of BG dye found in green paper towels (which are frequently used in hospitals, factories, and other facilities) involves transdermal penetration of the skin, even during brief exposure intervals (30–300 s) [17]. Similar effects were obtained for the consumption of fish contaminated with this dye, due to its illicit use in aquaculture [17]. Despite being hazardous to aquatic organisms, BG is easily integrated into aquatic systems due to its high solubility (100 g/L) [15,18]. Moreover, its extreme volatility pollutes the atmosphere, and its decomposition products (sulfur and nitrogen oxides, as well as carbon dioxide) are damaging to the environment [13,14,15,18]. Thus, removing this dye from wastewater has become a major challenge.

In the last few decades, different treatment technologies have been performed in order to remove BG dye from aqueous media, such as biological [19] and oxidative [20] processes, electrocoagulation [21], sono-catalytic degradation [22], membrane separation [23], and adsorption [7]. Weighing the advantages and limitations of the mentioned methods, absorption on the solid surface of the dye is one of the most effective methods in terms of simplicity and low costs [10]. In addition, for the adsorption process of BG to become economically feasible, researchers have focused on biodegradable, renewable, sustainable, easily available, and low-cost adsorbents, such as guava seeds [5], banana peels [7], kaolin [10], bagasse fly ash [12], sawdust [13], rice straw [15], medlar nucleus [17], Saklıkent mud [18], cashew nut shell [24], or acorn-based [25] materials. The adsorption technique has also been shown to be remarkable in the removal of other dye pollutants from aqueous solutions [4,8,26,27,28]. For example, environmentally friendly modifications to lignocellulosic materials such as walnut shells resulted in improved adsorption capabilities of two other cationic dyes with expanded medicinal applications (crystal violet and methylene blue) [26]. Furthermore, lignin-based magnetic biochar demonstrated significant Congo red removal efficiency in simulated wastewater [27], as well as excellent selective adsorption capability for methylene blue in binary, ternary, and quaternary dye systems [28]. However, for the treatment of contaminated waters, biosorbents derived from agricultural waste represent a valuable, affordable, and ecological alternative [29]. Pistachio (*Pistacia vera* L.) is considered one of the most valuable agricultural products from an economic standpoint in countries such as Iran, the US, Turkey, and Syria, which are responsible for almost 90% of the worldwide production [30,31]. The global demand for pistachio is increasing, mainly due to its beneficial impacts on human health (rich in nutrients and bioactive compounds) [32,33]. Considering their wide production and the shell/nut ratio of about 45%, large amounts of organic waste biomass are usually discharged by the processing industries to landfills (around 30 million tons/year) [8,31]. Furthermore, the chemical composition of pistachio shells, which contains abundant functional groups primarily provided by cellulose (40%), hemicellulose (25%), and lignin (16%) [8,34], can easily provide high added value and improved properties for dye adsorption processes.

The synthesis of sulfuric-acid-based activated carbon using pistachio shells was studied for methylene blue and brilliant green adsorption [35]. Due to its high adsorption capacity and increased surface area, activated carbon is one of the most widely utilized adsorbents; however, its high cost makes this technique uneconomical for commercial applications [10]. In this context, this study aimed to create a novel, simple, cost-effective, and environmentally friendly modified adsorbent based on pistachio shells for the removal of carcinogenic brilliant green from aqueous solutions. As far as we know, this is the first study to report the adsorption of brilliant green on a pistachio shell biosorbent obtained through simple grinding and mercerization processes. In addition to the characterization of the biosorbent from a physicochemical point of view, this study also reports the optimization of the separation process, as well as molecular docking simulations (to disclose the adsorption mechanism). Moreover, the thermodynamic parameters of adsorption were determined using two approaches: the classical one and the one based on the modified Langmuir isotherm proposed by Azizian et al. [36]. Both approaches take into account the saturation concentration of the investigated sorbate and lead to the same conclusions regarding the thermodynamics of the process. 

## 2. Results and Discussion

### 2.1. Modification of Pistachio Shells by Mercerization (PS_NaOH_)

To provide a cost-effective and practical solution for removing harmful cationic brilliant green dye from wastewater, pistachio shell waste was employed. This material was chosen for two reasons. First, large amounts of biomass are commonly discarded as agro-waste, leading to significant environmental concerns [31]. Second, pistachio shells possess diverse functional groups, such as cellulose (hydroxyl) and lignin content (hydroxyl, carbonyl, metoxyl, and carboxyl [34]). These functional groups make surface modification possible, which in turn can enable the acquisition of new properties that are suitable for cationic dye absorption procedures [37].

The alkaline treatment of pistachio shells was selected to improve the adhesive properties of the surface, providing a rough topography as a result of the removal of natural and artificial impurities from the surfaces of the pistachio shells [38]. Furthermore, this method is commonly used to activate cellulose by immersing the lignocellulosic biomass in NaOH solutions with concentrations ranging from 5 to 7% [39]. In this study, a concentration of 5% (NaOH) was used to reduce the environmental impact; also, the mercerized pistachio shells (PS_NaOH_) were carefully washed with water to eliminate the excess of NaOH. In contrast to other chemical agents, the use of NaOH as a surface modification agent also has benefits, including lower corrosion capacity, a lower environmental impact during its life cycle, and economic viability [40].

### 2.2. Characterization of PS_NaOH_ Biosorbent

**Figure 1:**
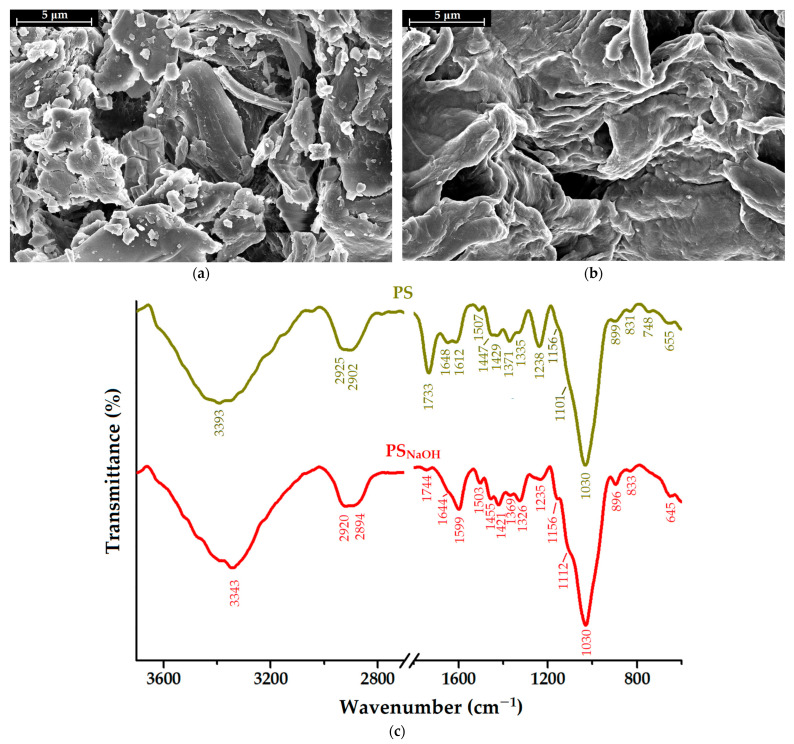
SEM images on the surface of (**a**) raw pistachio shell (PS) and (**b**) alkali-treated pistachio shell (PS_NaOH_); (**c**) FTIR spectra of unmodified and alkali-treated pistachio shells (PS and PS_NaOH_, respectively).

The surface morphological characteristics of the untreated and alkali-treated pistachio shells (PS and PS_NaOH_, respectively) were assessed through scanning electron microscopy (SEM), as shown in Figure 1a,b. A heterogeneous surface characteristic of lignocellulosic materials was visible in the untreated PS sample, as illustrated in Figure 1a. Along with the rough texture of the PS sample, the presence of irregularly shaped impurities was noticed. These have been assigned mainly to the crushing and grinding process of pistachio shells, given that similar outcomes were also observed for walnut shells subjected to this process [26]. By comparison with the raw PS sample, the SEM image of PS_NaOH_ (Figure 1b) reveals the ability of the NaOH to remove the solid impurities from the biosorbent surface. Moreover, the alkali treatment of pistachio shells led to a smoother, wavy surface with the exposure of open pores, which are expected to increase the adsorption capacity of organic molecules.

FTIR spectroscopy was used to investigate the structural changes at the surface of the PS_NaOH_ biosorbent induced by the alkali treatment. As depicted in Figure 1c, the PS_NaOH_ biosorbent’s spectrum was analyzed by comparison to the unmodified pistachio shell’s (PS) spectrum. Due to their structural complexity, both spectra have been represented with breaks to highlight the two characteristic regions, namely the OH and CH stretching vibrations in the region of 3700–2700 cm^−1^ and the “fingerprint” region in the range of 1800–600 cm^−1^. The corresponding assignments for the absorption bands are detailed in Table S1. In brief, the spectrum of raw PS shows distinctive absorption bands, which indicate the presence of cellulose, hemicellulose, and lignin components [40,41,42,43,44]. As a consequence of the alkaline treatment, a series of spectral changes for PS_NaOH_ have been observed, as shown in Figure 1c. As expected, the partial dissolution of lignin and hemicellulose occurred, as proven by the reduction in intensity and shifting of characteristic bands (1744 cm^−1^ and 1235 cm^−1^). This can be explained by the cleavage of the aliphatic side chains in lignin and the breakage of the C–O–C bonds between the two monomers during hemicellulose hydrolysis in an alkaline solution [43]. Moreover, it was noted that the intensities of the peaks at 1421 cm^−1^ and 896 cm^−1^ had increased, indicating the higher content of cellulose in the treated samples. Similar findings were made when shea nut shells were alkali-treated [45]. Moreover, the increase in the intensities of the hydroxyl and carbonyl functionalities at the biosorbent surface (3343 cm^−1^ and 1030 cm^−1^) is expected to improve the adsorption capacity of the PS_NaOH_ biosorbent.

The water vapor sorption capacity (W%) of the PS_NaOH_ sample was slightly higher by comparison with the PS sample (as given in Table S2). In addition, the Brunauer–Emmett–Teller kinetic (BET) and the Barrett–Joyner–Halenda (BJF) models were applied to calculate the specific surface area, the weight of water forming a monolayer, and the average pore size, respectively (following a protocol described in a previous work [46]). As shown in Table S2, the alkali treatment of pistachio shells led to a specific surface area of 253.2 m^2^/g and 323.5 m^2^/g for PS_NaOH_ and raw PS materials, respectively. However, one may say that the NaOH treatment of pistachio shells led to greater surface areas when compared with pistachio shells treated with H_2_O_2_ and NaOH or with HCl and HClO_4_ [40]. In addition, the average pore size was observed to increase after NaOH treatment from 1.210 nm to 1.563 nm (Table S2). Besides these outcomes, the alkaline treatment has the benefit of cleaning the pistachio shells’ surfaces of contaminants, which qualifies PS_NaOH_ for use in environmental applications.

### 2.3. Adsorption Studies of Brilliant Green onto PS_NaOH_ Biosorbent

#### 2.3.1. Adsorption Kinetics and Isotherms

Brilliant green (BG) cationic dye revealed three absorbance wavelengths (318, 426, and 624 nm, respectively), as highlighted by Figure S1a in the Supplementary Materials. To ensure the accuracy of the data, the adsorption investigations employed the band at 624 nm. Thus, the concentration of BG in the solution was measured based on the calibration curve depicted (Figure S1b).

**Figure 2:**
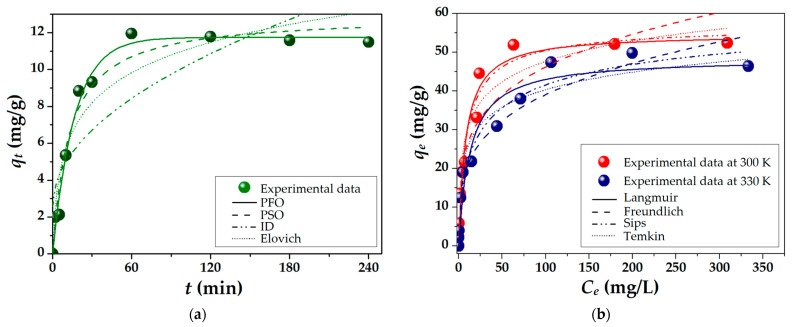
Adsorption of brilliant green (BG) onto PS_NaOH_ biosorbent: kinetic (**a**) and isotherm (**b**) studies (experimental data and mathematical models).

The BG adsorption kinetics onto the PS_NaOH_ biosorbent were evaluated, taking into consideration the influence of the contact time *t* (min) upon the adsorption capacity *q_t_* (mg/g). Thus, Figure 2a shows an increase in adsorption capacity over time, corresponding to three stages [47]. The first stage is characterized by rapid adsorption (in the first 30 min), mainly due to the large number of available sites on the PS_NaOH_ surface. With the depletion of active adsorption sites, the second stage begins and is represented by a slower adsorption process (between 30 and 120 min). The BG molecules try to diffuse into the pores and are gradually adsorbed by the inner pores until equilibrium is established (>120 min). The plateau zone at equilibrium is achieved in this final stage due to the saturation of the biosorbent’s reactive groups with BG molecules. A similar trend was observed by others when investigating BG adsorption onto chemically modified areca nut husk [48] and raw soybean milk residues [49].

To investigate the rate of adsorption and better understand the adsorption mechanism, pseudo-first-order (PFO), pseudo-second-order (PSO), intra-particle diffusion (ID), and Elovich kinetic models were employed (Figure 2a). The characteristic non-linear equations and parameters are given in Table S3 (Supplementary Materials). Furthermore, the chi-squared (*χ*^2^) statistical test was used to determine the agreement between predicted and experimental data. Given the smallest value of *χ*^2^ (Table S3) and the observations from Figure 2a, it is possible to conclude that the PFO model best describes the adsorption kinetics of BG onto the PS_NaOH_ biosorbent. The theoretical equilibrium adsorption capacity (*q_e_*^(calc)^ = 11.74 mg/g) estimated by the PFO model is closest to the experimental result (*q_e_*^(obs)^ = 11.49 mg/g) (see Table S3 in Supplementary Materials). These findings suggest that the adsorption mechanism of BG onto the PS_NaOH_ biosorbent is not predominantly based on diffusion (ID) [50].

The isotherm experiments were carried out at two temperatures (300 and 330 K) and represent the adsorption capacity at equilibrium (*q_e_*, mg/g) as a function of the concentration of BG dye at equilibrium (*C_e_*, mg/L), as illustrated in Figure 2b. As a result of the kinetic study (Figure 2a), the contact time was set at 240 min to ensure the adsorption equilibrium of the process. According to experimental isotherm data, the adsorption capacity at equilibrium (*q_e_*) decreased with temperature, changing from 52.42 mg/g (at 300 K) to 46.42 mg/g (at 330 K). This might be attributed to the higher kinetic energy of the BG molecule when the temperature rises, which can reduce the electrostatic attraction and separate the solute from the biosorbent’s surface [47]. These findings support the previously discussed kinetic results, corroborating that the increment in temperature does not cause the dye molecules to diffuse more inside the pores of the biosorbents. Other studies’ outcomes revealed the opposite temperature dependency behavior [48,49]. However, the greater adsorption capacities of BG dye at moderate temperatures (closer to those found in the environment) may be advantageous when used in wastewater treatment applications.

To further explore the adsorption process of BG cationic dye on the PS_NaOH_ biosorbent, the experimental data were interpolated by the Langmuir, Freundlich, Sips, and Temkin isotherm models, as plotted in Figure 2b. The non-linear equations of these models and the obtained isotherm parameters are detailed in Table S4 (Supplementary Materials). According to the calculated chi-square (χ^2^) values (see Table S4), the Sips isotherm model provided the best accuracy of data interpolation. Given that the Sips model combines the Langmuir (homogenous monolayer adsorption) and Freundlich (heterogeneous multilayer adsorption) models [51], it can be inferred that the BG molecules were adsorbed on the PS_NaOH_ surface in both manners, i.e., in homogeneous and heterogeneous layers. 

In addition, the computed values of the *R_L_* separation factor (0 < *R_L_* < 1) and of the *n_F_* Freundlich isotherm constant (2 < *n_F_* < 10) support the assertion of a favorable adsorption process (see Table S4 in Supplementary Materials) [48]. However, the values for the constants of Langmuir (*K_L_*), Freundlich (*K_F_*), and Temkin (*K_T_*), given in Table S4, are decreasing with increasing temperature, confirming the presence of a stronger interaction and improved affinity between the biosorbent surface and BG molecules at a lower temperature (of 300 K). To elucidate the adsorption mechanism (physical, ion exchange, or chemisorption [52]), the Dubinin–Radushkevich (D-R) model was applied, and the mean free energy (*E_S_*, kJ/mol) was calculated (Table S4). Given the values of *E_S_* ranging between 8 and 16 kJ/mol (~13 kJ/mol), it can be concluded that the adsorption process of BG cationic dye onto the PS biosorbent is mainly reliant on an ion-exchange mechanism between the surface acidic functional groups (e.g., −COO^−^) of the adsorbent and positively charged nitrogen atoms from the brilliant green cationic dye.

**Figure 3:**
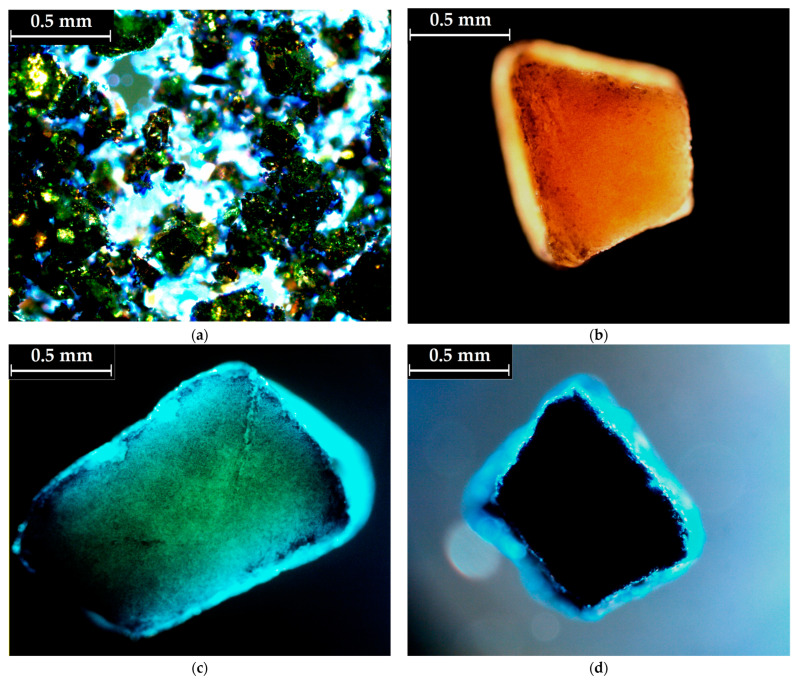
Polarization light images of (**a**) brilliant green solid particles (crystals); (**b**) non-loaded PS_NaOH_ grain; PS_NaOH_ grain after BG absorption from aqueous solutions with initial concentrations of (**c**) *C*_0_ = 10 mg/L and (**d**) *C*_0_ = 500 mg/L, respectively (*t* = 240 min).

The increased adsorption capacity of the PS_NaOH_ biosorbent with increasing concentrations of BG cationic dye in the aqueous solution was evidenced by polarized light microscopy (PoLM). As a triphenylmethane dye, brilliant green (Figure 3a) is known for its use as a fluorochrome in biological molecular labeling [53]. By comparison with the non-loaded (pristine) PS_NaOH_ biosorbent (Figure 3b), we observed the uniform adsorption of the BG dye onto the surface of the biosorbent (Figure 3c,d) after performing the isotherm study. One aspect to be noted is that after submerging the biosorbent in low concentrations of BG solution (10 mg/L for 240 min), the dye’s absorption was reduced, but light could still travel through the sample (Figure 3c). On the other hand, immersion in a concentrated solution with 500 mg/L of BG dye resulted in enhanced adsorption capacity (mg/g), which blocked the penetration of light (Figure 3d).

In addition, a comparative literature review on the maximum adsorption capacities of brilliant green (BG) on different lignocellulosic materials was carried out. As shown in Table 1, higher adsorption capacities (80–250 mg/g) were obtained for activated-carbon-derived materials from guava seeds [5], pistachio shells [35], and cashew nut shells [24]. However, in recent decades, researchers have concentrated on replacing expensive activated carbons with low-cost modified lignocellulosic materials. Thus, cost-effective modifications were approached (cleaning with distilled water, hot water treatment, NaOH, Na_2_CO_3_, or HCl treatment), yielding relevant adsorption capacities ranging from 18 to 59 mg/g [13,15,47,54,55,56]. Among these, sawdust from Indian Eucalyptus wood treated with NaOH had the highest value of BG adsorption (58.48 mg/g), followed by the PS_NaOH_ biosorbent employed in this investigation and similarly treated (54.74 mg/g). These findings support the use of PS_NaOH_ for the efficient removal of BG from aqueous solutions.

#### 2.3.2. Thermodynamic Parameters

Assessing the principal thermodynamic parameters (such as Gibbs free energy, enthalpy, and entropy) provides useful information regarding the energetic changes that occur during the adsorption [26,57,58,59,60,61]. In order to ascertain these thermodynamic parameters, it is necessary to take into consideration the adsorption equilibrium constants at a minimum of two distinct levels of temperature (e.g., 300 and 330 K). 

In this study, the adsorption equilibrium constant (*K_ad_*) and consequently the thermodynamic parameters were estimated on the basis of two approaches. In the first approach, the adsorption equilibrium constant (*K_ad_*) was approximated assuming the Langmuir equilibrium parameter *K_L_*, which was converted from the (L/mg) unit to the (L/mol) unit according to Equation (1) [26]:

(1) $$K_{L}\left(\frac{L}{\mathrm{mol}}\right)=K_{L}\left(\frac{L}{\mathrm{mg}}\right)\times 1000\left(\frac{\mathrm{mg}}{g}\right)\times M_{w}\left(\frac{g}{\mathrm{mol}}\right)$$

where *M_w_* (g/mol) is the molecular weight of the pollutant subjected to adsorption (i.e., BG dye). Afterward, the dimensionless adsorption equilibrium constant (*K_ad_*) was estimated in conformity with Equation (2) [26,58]:

(2) $$K_{ad}=K_{L}\left(\frac{L}{\mathrm{mol}}\right)\times C_{ref}\left(\frac{\mathrm{mol}}{L}\right)\times \frac{1}{\gamma }$$

where γ represents the activity coefficient ascertained on the basis of the ionic strength [36,58,60] and *C_ref_* denotes the molar concentration of the reference state. Commonly, in the classical approach, the concentration of pollutant or adsorbate (in the reference state) is considered to be 1 M (i.e., *C_ref_* = 1 mol/L) [58]. Hence, when *C_ref_* = 1 M, the adsorption equilibrium constant *K_ad_* (dimensionless) is numerically identical to the Langmuir constant *K_L_*. However, there are real situations in which the reference concentration of 1 M cannot be used, because the water solubility of the considered pollutant is less than 1 M. In this particular situation, the reference concentration is approximated to the saturated solution (*C_S_*) [36]. For example, the water solubility of the brilliant green (BG) dye is about 100 g/L (0.2072 mol/L). Thus, in the first approach, we calculated the adsorption equilibrium constant (*K_ad_*) by using Equation (2) and considering the saturated solution as the reference concentration (that is, *C_ref_* = *C_S_*, mol/L). Regarding the activity coefficient (γ), this was calculated by the Davis relationship [58].

In the second approach, the adsorption equilibrium constant (*K_ad_*) was considered to be equal to the dimensionless equilibrium parameter *K_ML_* from the revised Langmuir isotherm model (i.e., *K_ad_* = *K_ML_*). This revised Langmuir isotherm model was proposed and validated in 2018 by Azizian and co-workers [36], and the revised equation can be expressed as

(3) $$q_{e}=\frac{q_{m}K_{ML}C_{e}}{\left(C_{S}-C_{e}\right)+K_{ML}C_{e}}$$

where *q_e_* (mg/g) is the adsorption capacity at equilibrium, *q_m_* (mg/g) is the model parameter representing the maximum adsorption capacity as a monolayer, *C_e_* (mg/L) is the concentration of the pollutant (or adsorbate) at equilibrium, *C_S_* (mg/L) is the solubility of the pollutant (or adsorbate) in water (a saturated solution) and *K_ML_* (dimensionless) is the modified Langmuir constant representing the ratio between the elementary rate constants of adsorption (*k_a_*) and desorption (*k_d_*)—that is, *K_LM_* = *k_a_*/*k_d_*. The key difference between the modified Langmuir constant *K_LM_* and the classical Langmuir constant *K_L_* relies on the fact that *K_LM_* is a dimensionless quantity, whereas *K_L_* has specific units (L/mg) [36]. Therefore, the *K_LM_* constant can be directly employed in thermodynamic calculations, without additional mathematical transformations [36].

Thus, two approaches, (1) *K_ad_* = *K_L_* × *C_S_* × $\gamma^{-1}$ and (2) *K_ad_* = *K_ML_*, were further used to assess the thermodynamic parameters. Consequently, the changes in the Gibbs free energy of adsorption (Δ*G*) were computed as [26,36,58,60]

(4) $$\Delta G=-R_{g}T\;ln\left(K_{ad}\right)$$

where *K_ad_* is the equilibrium constant of adsorption (dimensionless), *T* is the absolute temperature (*K*), and *R_g_* is the universal gas constant (*R_g_* = 8.314 J/(K∙mol)). The variation in the enthalpy (Δ*H*) for the adsorption process was calculated by considering the isochore equation of Van’t Hoff, which may be expressed by Equation (5) [60]:

(5) $$\Delta H=\frac{-R_{g}ln\left(\frac{K_{ad,T_{2}}}{K_{ad,T_{1}}}\right)}{\frac{1}{T_{2}}-\frac{1}{T_{1}}}\;,$$

where *T*_1_ and *T*_2_ are two different values of temperature; consequently, $K_{ad,T_{1}}$ and $K_{ad,T_{2}}$ are adsorption equilibrium constants established for the corresponding temperatures. The modification of the entropy (Δ*S_ad_*) was assessed using the following thermodynamic equation [36,58,59]:

(6) $$\Delta S=\frac{\Delta H_{ad}-\Delta G_{ad}}{T},$$

The computed values of the thermodynamic parameters resulting from the two approaches are reported in Table 2. Both approaches revealed similar results, i.e., the calculated thermodynamic parameters by different methods were close in value (Table 2). It should be mentioned herein that the calculations according to the second approach (*K_ad_* = *K_ML_*) were faster since there was no need to compute the ionic strength and the activity coefficient separately. As summarized in Table 2, the negative values for the Gibbs free energy (Δ*G_ad_* < 0) indicated that the studied adsorption processes were spontaneous (exergonic). Moreover, the Δ*G_ad_* values less than −20 kJ/mol might suggest the presence of the ion-exchange phenomenon [61]. The calculated values of the enthalpies were found to be negative (Δ*H_ad_* < 0), indicating the exothermic nature of the studied adsorption processes. As reported in Table 2, the entropy change was positive (Δ*S_ad_* > 0) for all cases, highlighting the increase in randomness (at the solid–liquid interface) that contributed to the spontaneity of the process.

The revised Langmuir isotherm model (given by Equation (3)) was used to interpolate the experimental data obtained in the isotherm study (Section 2.3.1) and the isotherm curves are evidenced in Figure S2, while the corresponding parameters are detailed in Table S5 in the Supplementary Materials. As a result, the theoretical values for the maximum adsorption capacity (*q_m_*, mg/g) obtained by this approach are very close to those obtained by the classical Langmuir approach. However, it should be mentioned that the revised Langmuir isotherm model provides better data interpolation (smaller chi-square (χ^2^) values) for experiments conducted at low temperatures (e.g., 300 K).

#### 2.3.3. Design of Experiments (DoE) for Data-Driven Modeling and Optimization

The effectiveness of an adsorption process in wastewater treatment is largely determined by the optimal conditions [62]. In this study, the synergetic effect of two key factors, influencing the adsorption performance, was explored to establish the optimal conditions, i.e., (1) sorbent dose (*SD*, g/L) and (2) initial pollutant concentration *C*_0_ (mg/L). For adsorption process optimization, the design of experiments (DoE) and the response surface methodology (RSM) approach were adopted [63,64]. Brilliant green dye (BG) was employed as a target organic pollutant dissolved in the contaminant water, and the adsorption process was investigated in a systematic way. In this respect, a central composite design (CCD) of the rotatable type was applied for experimentation (Table 3). As detailed in Table 3, the operating variables (factors) are reported in terms of actual values (*SD* and *C*_0_) and coded values (*x*_1_ and *x*_2_). The experimental design listed in Table 3 involved 11 experimental runs. For each run, both factors (*SD* and *C*_0_) were varied simultaneously, recording the process response (*Y*,%). The central runs (9–11) from CCD (Table 3) were carried out at the midpoints of factor intervals to assess the reproducibility of the experiment. Thus, the reproducibility test was conducted in triplicate, as reported for runs 9, 10, and 11 (Table 3), which were carried out in the center of the experimental region. According to the reproducibility assay (runs 9, 10, and 11), the average value for the removal efficiency was equal to 93.68 ± 0.47%, which corresponded to an adsorption capacity of 33.48 ± 0.18 mg/g. Hence, the reproducibility error was less than 0.55%.

Based on the numerical data given in Table 3 (experimental matrix), a mathematical model was built using the multiple regression method [63,64]. In this regard, the multiple regression computations were performed using the Design Expert (v.10) program. The developed model, with two variables, is of the polynomial type and contains the following terms: (1) main (linear) effects, (2) curvature (quadratic), and (3) pairwise (two-variable) interaction. The model can be expressed in terms of coded variables (*x*_1_ and *x*_2_) as follows:

(7) $$\hat{Y}=39.68+20.33x_{1}-16.37x_{2}+13.53x_{1}x_{2}-16.07x_{1}^{2}-5.93x_{2}^{2}$$

$$\mathrm{subjected}\;\mathrm{to}:-1.414\leq x_{j}\leq 1.414;\;\left(j=1,2\right)$$

The resulting polynomial model (Equation (7)) was checked, regarding whether it was significant, by using the statistical test based on the analysis of variance (ANOVA) [63]. The main ANOVA statistical estimators are given in Table S6. As a result, the F-value of 26.51 and a small *p*-value of 0.0013 indicated a significant model from a statistical point of view. Consequently, the model can be used to navigate the design space through simulations. The value of the multiple correlation coefficient *R*^2^ (0.963) disclosed that about 96% of the variability in the sum of squares could be explained by the factors under consideration. After applying the mathematical substitution technique, the final empirical model with natural factors can be written as follows:

(8) $$\hat{Y}=40.56+32.15\times SD-0.26\times C_{0}+0.07\times SD\times C_{0}-4.02\times SD^{2}-5.39\times 10^{-4}\times {C_{0}}^{2}$$

$$\mathrm{subjected}\;\mathrm{to}:1.17\leq SD\leq 6.83\;\left(g/L\right);\;8.6\leq C_{0}\leq 291.4\;\left(\mathrm{mg}/L\right)\;$$

**Figure 4:**
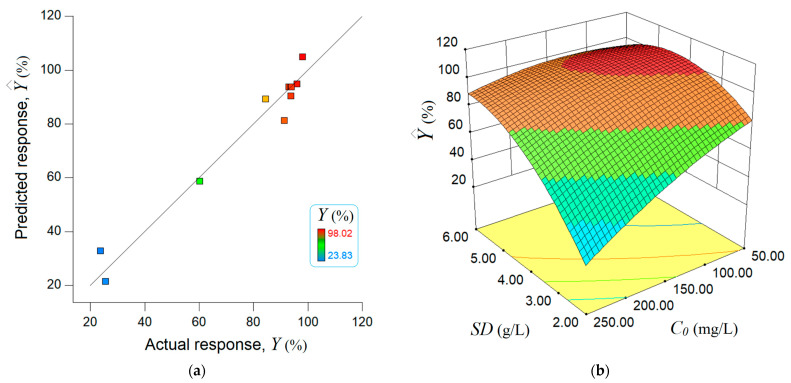
Data-driven modeling outcomes given by RSM: (**a**) agreement between experimental data and model predictions; (**b**) response surface of the estimated removal efficiency ($\hat{Y}$, %) dependent on the experimental factors, i.e., sorbent dose *SD* and initial pollutant concentration *C*_0_.

The estimations given by the data-driven model are shown in Figure 4. As highlighted in Figure 4a, there is reasonable agreement between the experimental data (actual response) and model estimations, since the data are scattered around the bisector (45° straight line). Figure 4b reveals that the main effect of the initial concentration (*C*_0_) factor is negative, while the main effect of the *SD* factor is positive with respect to the estimated response ($\hat{Y}$, %). In other words, the greater the *C*_0_ factor, the lower the estimated response. In turn, as the sorbent dose factor (*SD*) increases, the estimated response becomes higher (Figure 4b). The quadratic effects of both factors induce curvature in the response surface. In addition, there is an interaction effect between factors *SD* and *C*_0_. In accordance with this two-factor interaction effect, the influence of the *SD* factor is more noticeable at higher values of the initial concentration of BG dye. Instead, the effect of the *C*_0_ factor is more pronounced at smaller amounts of sorbent (Figure 4b).

Lastly, the constructed mathematical model was employed for process optimization. To this end, the numerical optimization was carried out by means of the direct search algorithm included in the Design Expert program. The model-based optimization results suggested the following optimal conditions: *SD* = 4.0 g/L and *C*_0_ = 10.1 mg/L. For these optimal conditions, the computed response was equal to $\hat{Y}$ = 104.98% (the predicted value), while the observed removal efficiency (recorded after 120 min of contact time) was Y = 98.78% (the actual value). The difference of about 6.20% represented the residual error between the model and the experiment. Note that the actual value of the response (98.78%) determined for the optimal conditions was the highest value compared to any value reported in Table 3. 

Following the determination of the optimal adsorption conditions for BG on the PS_NaOH_ biosorbent, the influence of the pH on the removal efficiency of BG was evaluated. Lower removal efficiency values were found at more acidic or alkaline pH levels, as indicated in Figure S3 (Supplementary Materials), as compared to the maximum value corresponding to the natural pH ≈ 6 (used in all the experiments by dissolving BG powder in distilled water). Once the pH is lowered (towards pH 2), additional H^+^ ions compete for the available adsorption sites (negatively charged) with the positively charged BG molecules, resulting in a decrease in dye removal efficiency [28]. By contrast, more adsorption sites (negatively charged) become accessible as the pH of the solution rises. These adsorption sites produce an electrostatic attraction with the cationic dye molecules, increasing the removal efficiency. Such a scenario was observed at pH 6, followed by decreasing removal efficiency for pH > 8, when the opposite effect (electrostatic repulsion) might occur [28,48]. However, it should be mentioned that PS_NaOH_ exhibited excellent BG adsorption ability with removal efficiency higher than 90% in a wide pH range (4–8), similar to the wastewater pH range.

In addition, the influence of coexisting anions (Cl^−^, NO_2_^−^, and SO_4_^2−^), cations (Na^+^, Ca^2+^, and Fe^2+^), or organic matter (humic acid) on BG removal onto the PS_NaOH_ biosorbent was assessed [27,28]. As depicted in Figure S4 in the Supplementary Materials, the addition of different salts led to a decrease in BG removal efficiency from 98.87% (in the absence of supplemental ions) to values in the range of 34.52–86.57% (in the presence of coexisting ions) and to 88.08% in the presence of humic acid (Figure S4). However, PS_NaOH_ demonstrated good removal efficiency of BG in the presence of different ions (except CaCl_2_) and organic matter, highlighting its practical applicability in wastewater treatment.

### 2.4. Molecular Modeling and Docking

Molecular modeling is a pivotal tool for performing simulations at the atomistic level to explore conformational geometries and electronic structures, as well as to grasp intra- and intermolecular interactions.

Brilliant green (BG), also known under various synonyms (malachite green G, basic green 1, and emerald green) is an organic hydrogen sulfate salt having 4-{[4-(diethylamino)phenyl](phenyl)methylidene}-N,N-diethylcyclohexa-2,5-dien-1-iminium (C_27_H_33_N_2_^+^) as the counterion [65]. Computational results (by DFT) for the BG molecule (in its cationic form) are summarized in the Supplementary Materials (Figure S5 and Table S7).

**Figure 5:**
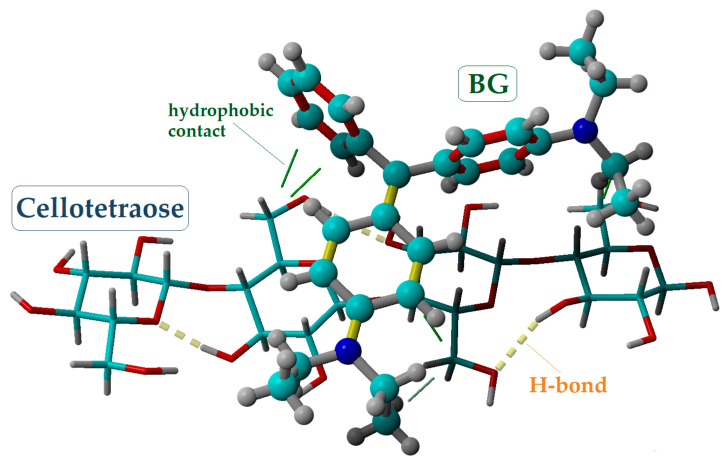
Molecular docking outcomes, showing the best pose of the docked complex between cellotetraose receptor and BG ligand, as well as their interaction mode; *E_b_* = −2.84 kcal/mol and *K_d_* = 8.27 mM.

**Figure 6:**
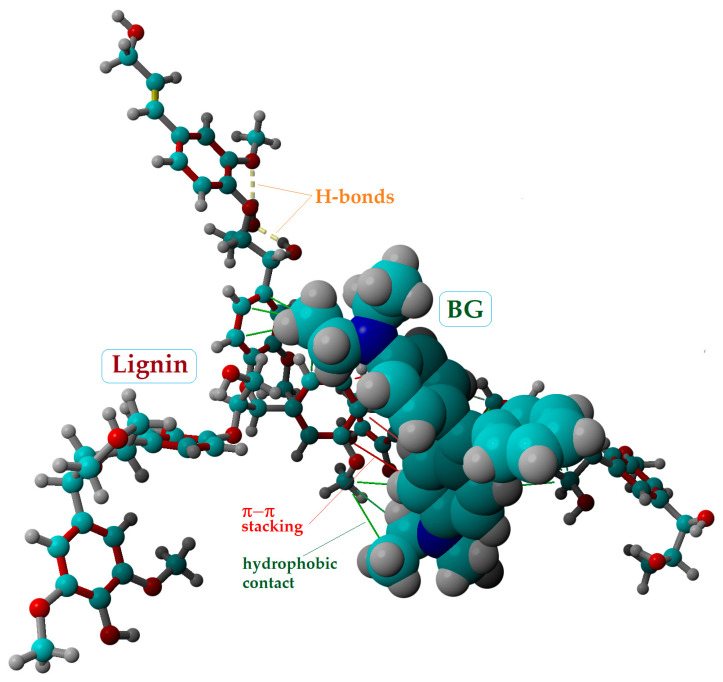
Molecular docking outcomes, showing the best pose of the docked complex between lignin receptor and BG ligand, and their interaction mode; *E_b_* = −4.59 kcal/mol and *K_d_* = 0.43 mM.

In the subsequent molecular modeling procedures, the BG molecule (cationic form) was considered the ligand for molecular docking computations. To this end, its optimized geometry (at the DFT level) was taken as the starting structure for these simulations. Likewise, for the molecular docking computations, we considered two receptors: (1) cellulose oligomer (cellotetraose) and (2) lignin. The outcomes of the molecular docking are illustrated in Figure 5 and Figure 6 for the cellotetraose receptor and lignin receptor, respectively. In these figures, the best poses of the docked complexes are given, detailing the intermolecular interactions between the ligand (BG) and receptors. As shown in Figure 5, the hydrogen bonds (H-bonds) are present inside the cellotetraose receptor (intramolecular interactions) and are rendered as dotted yellow lines. The intermolecular interactions between cellotetraose and the BG molecule rely on hydrophobic contacts (highlighted in Figure 5 as solid green lines). These hydrophobic contacts can be correlated with the physical adsorption (on the basis of van-der-Waals forces) of BG dye onto cellulose. Computational results revealed for the docked complex cellotetraose–BG a binding energy (*E_b_*) of −2.84 kcal/mol and a dissociation constant (*K_d_*) equal to 8.27 mM. The large value of the dissociation constant (*K_d_* = 8.27 mM) suggests that the interaction between the ligand and cellotetraose receptor is not strong and that the dissociation of the docked complex is possible.

For the second case, the docked complex lignin–BG is illustrated in Figure 6. Compared to the previous complex (cellotetraose–BG), the interaction between the lignin receptor and BG ligand was stronger, disclosing lower values for the affinity parameters (i.e., *E_b_* = −4.59 kcal/mol and *K_d_* = 0.43 mM). Moreover, molecular docking results indicated that, for this case, beyond the hydrophobic contacts, π–π stacking interactions can emerge between the lignin and BG (rendered in Figure 6 as red lines). These types of interactions (π–π stacking) refer to the presumptive attractive, non-covalent interactions (orbital overlap) between the π bonds of aromatic rings. The π–π interactions might be associated with chemisorption. It should be mentioned that the simulations also revealed intramolecular H-bond formation in the conformation of the lignin receptor (Figure 6).

In addition, the interaction energies between the ligand (BG) and receptors (cellotetraose and lignin) were calculated at the level of the YASARA force field in order to account for the contributions of the van-der-Waals and Coulomb forces. In this respect, the interaction energy (Δ*E*) between the ligand and receptor (relying on the molecular force field) was calculated according to [66]

Δ*E* = *E_COMPLEX_* − (*E_RECEPTOR_* + *E_LIGAND_*) (9)

where Δ*E* is the energy of interaction between the ligand and receptor, *E_COMPLEX_* denotes the potential energy of the docked complex, *E_RECEPTOR_* is the potential energy of the receptor, and *E_LIGAND_* is the potential energy of the ligand. Note that each term (from Equation (9)) includes both intramolecular and intermolecular contributions. The latter one involves two individual energy components for van-der-Waals (vdW) and Coulomb forces, which are responsible for distance-dependent attractive/repulsive interactions and electrostatic effects, respectively [66]. In general, the lower the interaction energy, the stronger the interaction between the ligand and receptor.

Table 4 gives the energies of intermolecular interactions between the BG dye (ligand) and both receptors. As reported in Table 4, the interaction between BG and cellotetraose is based principally on vdW forces (Δ*E_vdW_* = −9.00 kcal/mol). In turn, the electrostatic interactions for the cellotetraose–BG complex are minor (Δ*E_CL_* = −0.97 kcal/mol). For the second case of lignin binding with BG, the major role is attributed to electrostatic interactions (Δ*E_CL_* = −21.30 kcal/mol), whereas the vdW forces (Δ*E_vdW_* = −8.21 kcal/mol) were of secondary importance. The docking results for the lignin–BG system were in good agreement with the D-R isotherm results that suggested a mechanism of adsorption based on ion exchange (i.e., electrostatic interactions). The overall intermolecular interaction energy (Δ*E* = Δ*E_vdW_* + Δ*E_CL_*) was lower for the lignin–BG complex (Table 4), indicating that the ligand (BG dye) interacted more strongly with lignin compared to cellotetraose.

## 3. Materials and Methods

### 3.1. Materials

Brilliant green (BG) and sodium hydroxide ≥ 97.0% were purchased from Merck Chemical (Saint Louis, MO, USA). A stock experimental solution of BG cationic dye was prepared by using distilled water (concentration of 1 g/L). The pistachio shells (acquired from a local grocery store) were subjected to mechanical grinding using a Pulverisette 11 knife mill (Fritsch, Idar-Oberstein, Germany) after pre-washing with distilled water and drying at 378 K in a laboratory oven. Pistachio shells (PS) were further subjected to an alkaline treatment by being immersed in a 5% NaOH aqueous solution and magnetically stirred (150 rpm) for 48 h at room temperature. The final alkali-treated PS_NaOH_ biosorbent was produced after the grains were filtered, thoroughly rinsed with bi-distilled water (to remove excess sodium hydroxide), and dried at 338 K for 24 h in an oven. Grains with sizes between 0.5 and 1.5 mm were obtained (Figure S6 in the Supplementary Materials).

### 3.2. PS_NaOH_ Biosorbent Characterization

The surface morphological properties of the alkali-treated biosorbent were examined using a Quanta 200 Scanning Electron Microscope (Brno, Czech Republic). A structural investigation was performed using a Bruker Vertex 70 Fourier Transform Infrared Spectrophotometer (Ettlingen, Germany) in the range of 4000–600 cm^−1^ in attenuated total reflectance mode (2 cm^−1^ resolution and 64 scans). Brilliant green adsorption onto the PS_NaOH_ biosorbent was evidenced using a polarized optical microscope (Leica Microsystems, Wetzlar, Germany). The water vapor sorption capacities of the PS and PS_NaOH_ samples were determined in the dynamic regime by using IGAsorp fully automated gravimetric equipment (Hiden Analytical, Warrington, UK).

### 3.3. Adsorption of Brilliant Green onto PS_NaOH_ Biosorbent

Brilliant green adsorption onto the PS_NaOH_ biosorbent was employed by using an orbital shaker incubator, the Biosan ES-20/60 (Riga, Latvia). A Hitachi U-3900 dual-beam UV–VIS spectrophotometer (Hitachinaka, Japan) was used to measure the dye content in the aqueous solutions, using previously determined calibration curves and the detection of adsorption bands (318, 426, and 624 nm), as depicted in Figure S1.

Kinetic experiments were carried out in batch mode to determine the adsorption capacity of the sorbent over time (T = 300 K). Thus, 0.2 g of PS_NaOH_ biosorbent was introduced in 50 mL of 50 mg/L BG solution (orbital shaker, 180 rpm, 4 h). The dye concentration in the solution was determined by taking aliquots from the dye solutions at various predetermined contact times. The adsorption kinetics as a function of time were calculated according to Equation (9):

(10) $$q_{t}\left(\mathrm{mg}/g\right)=\frac{\left(C_{0}-C_{t}\right)V}{m\cdot 1000},$$

where *q_t_* (mg/g) is the amount of dye absorbed at time *t* (min); *C*_0_ and *C_t_* (mg/L) are the dye concentrations in the initial and final solutions (after contact time *t*), respectively; *V* (mL) is the volume of the immersion medium, and *m* (g) represents the weight of the PS_NaOH_ biosorbent.

The adsorption isotherms were measured at two different temperatures: 300 K and 330 K. As a result, for the same 0.2 g amount of biosorbent, the initial concentrations of the dye solutions ranged between 10 and 500 mg/L (contact time was set at 4 h, 180 rpm). The adsorption capacity at equilibrium (*q_e_*, mg/g) was calculated based on Equation (9) (by introducing the *C_e_* concentration at equilibrium instead of the *C_t_* concentration at time *t*).

The effect of coexisting anions (Cl^−^, NO_2_^−^, and SO_4_^2−^) and cations (Na^+^, Ca^2+^, and Fe^2+^) from different salt species was investigated in a 50 mL solution of 10 mg/L BG (with 200 mg/L of NaCl, CaCl_2_, NaNO_2_, or FeSO_4_ salt content and a 4 g/L sorbent dose). Similarly, the effect of organic matter was evaluated using humic acid (20 mg/L).

### 3.4. Optimization of the Adsorption Process

The adsorption process’ optimization was carried out by applying the design of experiments (DoE) and the response surface methodology (RSM) [63,64]. Brilliant green dye (BG) was used as a model organic pollutant and was dissolved in an aqueous medium. The experiments were done at a temperature of 300 K and at a naturally occurring pH of 6.0 ± 0.2. The adsorption performance was quantified via the color removal efficiency *Y* (response of the process), which was determined experimentally after a contact time of 120 min. The aim of the optimization was to maximize the color removal efficiency *Y* (%), which can be expressed as

(11) $$Y\left(\%\right)=\left(1-\frac{C_{t}}{C_{0}}\right)\times 100,$$

where *C*_0_ denotes the initial concentration of the BG dye (organic pollutant), and *C_t_* is the residual concentration of the BG dye determined after a contact time of *t* = 120 min. For modeling purposes, the sorbent dose (*SD*, g/L) and initial pollutant concentration *C*_0_ (mg/L) were investigated as the main factors and were transformed into coded (dimensionless) variables *x*_1_ and *x*_2_. This conversion was carried out to compare the effects of the factors using the same dimensionless scale. The mathematical relationships adopted for the conversion of actual factors into coded variables have been detailed by others [63,64].

### 3.5. Molecular Modeling of Brilliant Green Cationic Dye

The initial 3D conformer of BG, in its cationic form (C_27_H_23_N_2_^+^), was downloaded from the PubChem database [65] (PubChem CID 12449) and then subjected to molecular modeling at the level of density functional theory (DFT). The molecular modeling simulations were performed on a Dell Precision workstation T7910 with 32 CPU threads.

The quantum chemical calculations by the DFT method were carried out using the Gaussian16 software package [67]. The density functional with spherical atom dispersion terms APFD [68] was applied in this case by considering a split-valence (triple-zeta) basis set with polarization functions, i.e., APFD/6-311G(2d,p). The outcomes of DFT simulations were visualized and analyzed using the GaussView 6 program [69].

### 3.6. Molecular Docking

The molecular docking simulations were performed by using the AutoDock VINA algorithm [70] inbuilt into the YASARA-Structure program (v.20.8.23) for modeling and visualization [71,72]. The molecular conformation of the first receptor (cellulose oligomer) was optimized at the level of the YASARA force field, starting from the crystallographic data of cellotetraose already reported [73]. Regarding the reference structure of lignin (the second receptor), this was retrieved from the PubChem platform [34] (PubChem CID: 73555271), and then it was subjected to a minor modification by adding a moiety (–COO^−^) in order to simulate the presence of one carboxylic group. Subsequently, the conformation (3D structure) of this second receptor was subjected to energy minimization (for geometry optimization) at the level of the YASARA force field. Molecular docking simulations were carried out in the YASARA-Structure program environment. In this regard, for each system, a total of 100 docking poses were assayed at the level of molecular mechanics theory using the YASARA force field. In the course of the molecular docking simulations, the ligand (BG molecule) was treated as a flexible body, while both receptors were treated as rigid bodies. The parameters for the modeled structures were generated automatically by means of the algorithm “AutoSMILES” included in the YASARA-Structure program.

## 4. Conclusions

Pistachio shells (lignocellulosic waste) were subjected to grinding and alkaline treatment to obtain an efficient, low-cost biosorbent (PS_NaOH_). The morphological and structural modifications were explored via the SEM, FTIR, and PoLM characterization techniques. Brilliant green (BG) cationic dye was used in the adsorption experiments as a hazardous organic pollutant dissolved in water. The investigated adsorption process followed pseudo-first-order (PFO) kinetics. The experimental data on adsorption isotherms revealed a maximum adsorption capacity of 52.42 mg/g at 300 K. The Sips isotherm model provided the best accuracy of data interpolation. The values of the mean free energy of sorption (*E_S_*, resulting from the Dubinin–Raduschevich isotherm) were around 13.1 kJ/mol, indicating that the adsorption mechanism was mainly based on ion exchange. 

Two approaches were applied to assess the adsorption equilibrium constant (*K_ad_*) and, consequently, the thermodynamic parameters. Both methods revealed similar results regarding the values of thermodynamic parameters. For the approach relying on the revised Langmuir isotherm model, there is no need to compute the activity coefficient. The negative values for the Gibbs free energy (Δ*G_ad_* < 0) and for the enthalpy (Δ*H_ad_* < 0) indicated that the studied adsorption processes were spontaneous and exothermic. 

The process optimization based on the response surface methodology (RSM) indicated the optimal conditions for the adsorption of BG onto the PS_NaOH_ (i.e., *SD* = 4.0 g/L and *C*_0_ = 10.1 mg/L). The experimental results confirmed that under the optimal conditions established, the effective removal efficiency was *Y* = 98.78%, which was the maximum value observed in this study. 

The outcomes of the molecular docking computations revealed the possible intermolecular interactions between lignocellulosic receptors (cellulose oligomer or lignin) and the BG molecule. For the cellotetraose–BG docked complex, the interactions were based mainly on hydrophobic contacts (vdW forces). Instead, the lignin–BG docked complex was stabilized by hydrophobic contacts, π–π stacking, and electrostatic (Coulomb) interactions.

## Figures and Tables

**Table 1:** Comparison of the maximum adsorption capacities (*q_m_*) of different sorbent materials for BG cationic dye removal.

Sorbents Derived from Lignocellulosic-Based Biosorbents	Modifications	Maximum Adsorption Capacity (*q_m_*, mg/g)	Ref.
Activated carbon (AC) from acorn	Carbonization in argon atmosphere	2.01	[25]
Activated carbon (AC) from guava seeds	Chemical activation with phosphoric acid	80.50	[5]
Activated carbon (AC) from pistachio shells	Chemical activation with sulfuric acid	151.52	[35]
Activated carbon (AC) from cashew nut shells	Chemical activation with potassium hydroxide	243.90	[24]
Peanut shells	Washing with water	19.92	[54]
Watermelon peels	Hot water treatment	25.00	[55]
Rice straw	Washed with water	30.68	[15]
Areca nut husk	NaOH treatment	18.21	[47]
Sawdust from Indian Eucalyptus wood	NaOH treatment	58.48	[13]
*Bambusa Tulda*	Na_2_CO_3_ treatment HCl treatment Distilled water washing	41.67 31.25 32.25	[56]
Pistachio shells	NaOH treatment	54.74	This work

**Table 2:** Thermodynamic parameters for the adsorption of BG dye onto PS_NaOH_ biosorbent; thermodynamic calculations performed via two approaches.

Approach ^1^	*T* (K)	*K_ad_*	Δ*G_ad_* (kJ/mol)	Δ*H_ad_* (kJ/mol)	Δ*S_ad_* (J/K∙mol)
(1) *K_ad_* = *K_L_* × *C_S_* × $\gamma^{-1}$	300 K	1.779 × 10^4^	−24.411	−12.602	39.363
	330 K	1.124 × 10^4^	−25.592		39.364
(2) *K_ad_* = *K_ML_*,	300 K	1.236 × 10^4^	−23.502	−12.681	36.070
	330 K	7.786 × 10^3^	−24.584		36.069

^1^ *K_ad_* is the adsorption equilibrium constant, *K_L_* is the Langmuir equilibrium parameter (L/mol); *C_s_* denotes the concentration of the saturated solution; γ = 0.695; *K_ML_* is the dimensionless equilibrium parameter from the revised Langmuir isotherm model.

**Table 3:** Central composite design (rotatable type) adopted for experimentation of BG dye removal from aqueous solutions by adsorption using PS_NaOH_ as an adsorbent.

Run	Sorbent Dose		Initial Concentration of BG Dye		Removal Efficiency (Response), Determined after 120 Min of Contact Time
	Coded *x*_1_	Actual *SD*, g/L	Coded *x*_2_	Actual *C*_0_, mg/L	
1	−1	1.00	−1	50.0	91.40
2	+1	6.00	−1	50.0	96.12
3	−1	1.00	+1	250.0	25.57
4	+1	6.00	+1	250.0	84.42
5	−1.414	1.17	0	150.0	23.83
6	+1.414	6.83	0	150.0	93.88
7	0	4.00	−1.414	8.6	98.02
8	0	4.00	+1.414	292.4	60.25
9	0	4.00	0	150.0	93.15
10	0	4.00	0	150.0	93.84
11	0	4.00	0	150.0	94.06

**Table 4:** Energy of intermolecular interactions between BG cationic dye (ligand) and receptors (cellotetraose and lignin) calculated at the level of the YASARA force field.

Docking System (Ligand–Receptor)	Total Intermolecular: Δ*E =* Δ*E_vdW_ +* Δ*E_CL_* (kcal/mol)	van-der-Waals: Δ*E_vdW_* (kcal/mol)	Coulomb: Δ*E_CL_* (kcal/mol)
Cellotetraose–BG	−9.97	−9.00	−0.97
Lignin–BG	−29.51	−8.21	−21.30

## Data Availability

Not applicable.

## Supplementary Materials

The following supporting information can be downloaded at: https://www.mdpi.com/article/10.3390/molecules28104129/s1, Figure S1: (a) Determination of maximum absorbance wavelengths for brilliant green cationic dye; (b) Calibration curve graphical representation of at a wavelength of 624 nm (concentration range of 0–10 mg/L); Figure S2: Adsorption isotherm data fitted to the revised Langmuir isotherm model (Azizian approach [36]), showing the adsorption equilibrium established between the surface of pistachio shell adsorbent and brilliant green (BG) cationic dye; water solubility of BG dye is *C_S_* = 1×10^5^ mg/L.; Figure S3: Effect of pH: removal efficiency of brilliant green (BG) from aqueous solutions of pH 2 and 4 (adjusted with 0.1 M H_2_SO_4_) and pH 8 and 10 (adjusted with 0.1 M NaOH) compared to natural pH 6; optimal experimental conditions: *SD* = 4 g/L, *C*_0_ = 10 mg/L, *V* = 50 mL, *t* = 240 min); Figure S4: Removal efficiency of brilliant green (BG) from aqueous solutions in the presence of different salts (200 mg/L NaCl, CaCl_2_, NaNO_2_, or FeSO_4_), and in the presence of organic matter (20 mg/L humic acid); optimal experimental conditions: *SD* = 4 g/L, *C*_0_ = 10 mg/L, *V* = 50 mL, *t* = 240; Figure S5: Molecular structure and particularities of BG molecule estimated computationally at DFT/APFD/6-311G(2d,p) level of theory for ground state S_0_ (charge = +1, singlet): (a) optimized geometry and dipole moment; (b) frontier molecular orbitals (HOMO, LUMO); (c) partial atomic charges (Mulliken) distribution; (d) electrostatic potential (ESP) map. Min; Figure S6: PS_NaOH_ grains image obtained by optical microscope (Conrad USB, Wels, Austria); Table S1: FTIR spectra absorption band assignments for unmodified and alkali-treated pistachio shells (PS and PS_NaOH_, respectively); Table S2: Surface parameters of PS and PS_NaOH_ samples evaluated based on dynamic vapor sorption measurements: water vapor sorption capacity, final weight (*W*); average pore size (*p_s_*), surface area (*A*), and monolayer weight (*W_m_*); Table S3: Kinetic model equations and parameters* for BG dye adsorption onto PS_NaOH_ biosorbent (experimental conditions: T = 300 K, sorbent dose = 4 g/L, *C*_0_ = 50 mg/L); Table S4: Isotherm models (equations and parameters) for BG cationic dye adsorption onto PS_NaOH_ biosorbent (*t* = 240 min, sorbent dose = 4 g/L); Table S5: Parameters of the revised Langmuir isotherm model determined for BG dye adsorption onto PS_NaOH_ biosorbent (CS = 1 × 10^5^ mg/L (solubility for BG dye)); Table S6: Analysis of variance (ANOVA) for the multiple regression model $\hat{Y}\left(x_{1},x_{2}\right)$; Table S7: Summary of molecular and global reactivity descriptors for BG molecule (cationic form); descriptors computed by DFT method at APFD/6-311G(2d,p) level of theory.

## Sample Availability

Not available.
