# Supplementary material for: Adsorption of Brilliant Green Dye onto a Mercerized Biosorbent: Kinetic, Thermodynamic, and Molecular Docking Studies
Source: Molecules. 2023 May 16;28(10):4129. doi: 10.3390/molecules28104129 (PMC10222809; doi:10.3390/molecules28104129)
Supplement: Supplementary file 1 [file molecules-28-04129-s001.zip › molecules-2386037-supplementary.pdf]

molecules

Supplementary materials

Table S1. FTIR spectra absorption bands assignments for unmodified and alkali treated pistachio shells (PS and PS_{NaOH}, respectively).

FTIR assignements	Wavenumber (cm^{-1})		Ref.
	PS	PS_{NaOH}	
OH stretching vibrations from cellulose and lignin (free OH(6) and OH(2), O(2)H...O(6) and O(3)H...O(5) intramolecular hydrogen bonds, O(6)H...O(3) intermolecular hydrogen bonds in cellulose, intermolecular hydrogen bonds between lignin phenolic groups and their combinations with alcoholic groups)	3393	3343	
CH asymmetric stretching vibrations in lignin, cellulose and hemicellulose (aromatic methoxyl groups, methyl and methylene groups)	2925	2920	
CH symmetric stretching vibrations in lignin, cellulose and hemicellulose	2902	2894	
C=O stretching vibrations in hemicellulose and lignin (unconjugated ketone, carbonyl, and aliphatic groups)	1733	1744	
C=O stretching vibrations from aromatics skeletal in hemicellulose and lignin<br>OH bending vibrations associated with absorbed water in lignin or hemicellulose	1648	1644	
C=C aromatic stretching vibrations in lignin (syringyl)	1612	1599	
C=C aromatic stretching vibrations in lignin (guaiacyl)	1507	1503	[40-44]
C–H asymmetric deformation in lignin (methyl and methylene)<br>O-H in-plane bending	1447	1455	
C–H asymmetric deformation in $–OCH_3$	1429	1421	
CH bending in cellulose and hemicellulose	1371	1369	
CH_2 wagging in cellulose	1335	1326	
C-O, C-C, C=O and guaiacyl ring stretching in lignin and C-O vibration of acetyl groups in hemicellulose	1238	1235	
C–O–C asymmetric stretching in cellulose	1156	1156	
C–O stretching in secondary alcohols and aliphatic ether in cellulose<br>aromatic C–H in-plane deformation in lignin (typical for syringyl units)	1101	1112	
C–O and C–C stretching ring in cellulose and hemicelluloses	1030	1030	
C-C-O, C-O-C stretching of $\beta(1\rightarrow4)$- glycosidic linkages and C-H bending in cellulose	899	896	
aromatic sp^2 bending of (C-H) from lignin	831	833	
C-C skeleton vibration	748	-	
C-OH out-of-plane bending<br>O-C-O in-plane deformation	655	645	

Table S2. Surface parameters of PS and PS_{NaOH} samples evaluated based on dynamic vapors sorption measurements: water vapor sorption capacity, final weight (W); average pore size (p_s), surface area (A) and monolayer weight (W_m).

Biosorbent	W (%)	BET data		BJH model
		**Monolayer (W_m, mg/g)**	**Area (A, m^2/g)**	**Average pore size (p_s, nm)**
PS	19.58	92.15	323.5	1.210
PS_{NaOH}	19.79	72.12	253.2	1.563

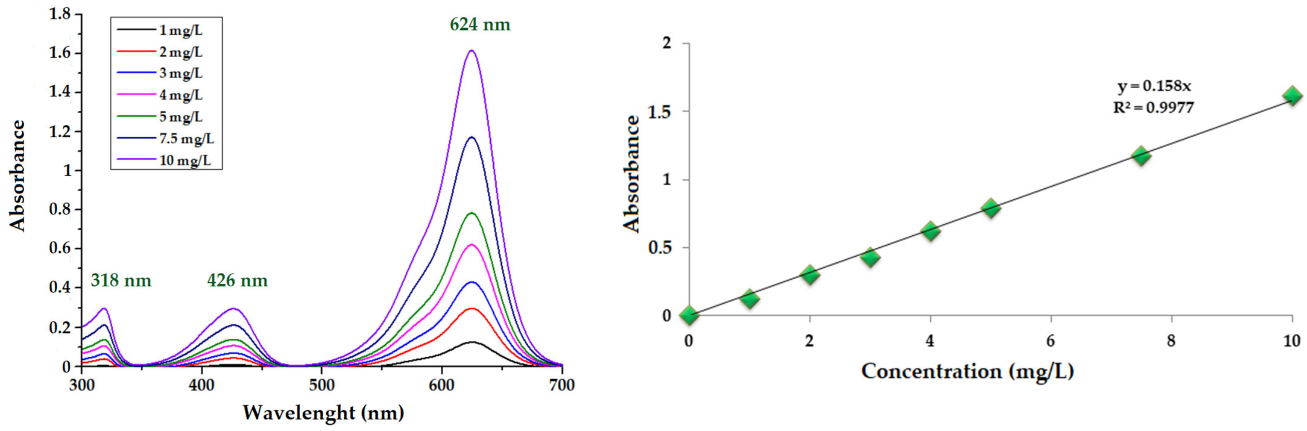

(**a**) (**b**)

Figure S1. (**a**) Determination of maximum absorbance wavelengths for Brilliant green cationic dye; (**b**) Calibration curve graphical representation of at a wavelength of 624 nm (concentration range of 0-10 mg/L).

Table S3. Kinetic models equations and parameters* for BG dye adsorption onto PS_{NaOH} biosorbent (experimental conditions: T = 300 K, sorbent dose = 4 g/L, C_0 = 50 mg/L).

Kinetic Model	Non-Linear Equation [1]	Kinetic parameters [2]	Experimental value of absorption capacity (mg/g)
**PFO**	$q_t = q_e(1 - e^{-k_1 t})$	q_e = 11.74 (mg/g)<br>$k_1 = 0.60 \times 10^{-1}$<br>$\chi^2$ = 0.28	$q_e^{(obs)}$ = 11.49 (mg/g)
**PSO**	$q_t = \frac{k_2 q_e^2 t}{1 + k_2 q_e t}$	q_e = 12.94 (mg/g)<br>$k_2 = 0.61 \times 10^{-2}$<br>$\chi^2$ = 0.74	
**ID**	$q_t = k_d\sqrt{t} + J$	k_d = 0.76<br>J = 2.58<br>χ^2 = 6.44	
**Elovich**	$q_t = \frac{1}{\beta} ln(1 + \alpha\beta t)$	α = 2.33<br>β = 0.41<br>χ^2 = 2.10	

[1] q_e and q_t are the amounts of BG dye adsorbed at equilibrium and at time t, respectively (mg/g); k_1 and k_2 are the pseudo-first-order and pseudo-second order rate constants; t is the contact time between the adsorbent and adsorbate (min); k_d is the intra-particle rate constant (mg/g · min$^{1/2}$) and J is the intercept of intraparticle diffusion kinetic model; β gives the number of available sites for adsorption and α is the initial adsorption rate (mg/ g· min) [46,49].

[2] Chi-square (χ^2) statistic test was calculated using Equation S1:

$$\chi^2 = \sum (q_e^{(obs)} - q_e^{(calc)})^2 / q_e^{(calc)}, \quad \textbf{(S1)}$$

where, $q_e^{(obs)}$ and $q_e^{(calc)}$ represent the observed (experimental) and the calculated (theoretical) values of adsorption capacity (mg/g).

Table S4. Isotherm models (equations and parameters) for BG cationic dye adsorption onto PS_{NaOH} biosorbent (t = 240 min, sorbent dose = 4 g/L).

Temperature	Isotherm models and parameters [1]				
	Langmuir	Freundlich	Sips [2]	Temkin	Dubinin-Raduschevich
	$q_e = \frac{q_m K_L C_e}{1 + K_L C_e}$	$q_e = K_F C_e^{1/n_F}$	$q_e = \frac{q_S K_S C_e{}^{1/nS}}{1 + K_S C_e{}^{1/nS}}$	$q_e = B_T\, ln(K_L C_e)$	$E_S = \frac{1}{\sqrt{2K_D}}$
**T = 300 K (27 °C)**	q_m = 54.744 (mg/g)<br>K_L = 0.123 (L/mg)<br>R_L = 0.116<br>χ^2 = 11.478	K_F = 15.138<br>n_F = 4.128<br>χ^2 = 49.770	q_s = 57.224<br>K_S = 0.158<br>n_S = 1.194<br>χ^2 = 11.320	K_T = 5.329<br>B_T = 7.591<br>χ^2 = 25.704	K_D = 2.901× 10^{-3}<br>E_S = 13.127 (kJ/mol)<br>r^2 = 0. 955
**T = 330 K (57 °C)**	q_m = 48.474 (mg/g)<br>K_L = 0.078 (L/mg)<br>R_L = 0.160<br>χ^2 = 20.330	K_F = 11.152<br>n_F = 3.673<br>χ^2 = 19.993	q_s = 64.045<br>K_S = 0.140<br>n_S = 1.785<br>χ^2 = 12.258	K_T = 4.928<br>B_T = 6.509<br>χ^2 = 18.040	K_D = 2.882 × 10^{-3}<br>E_S = 13.171 (kJ/mol)<br>r^2 = 0. 975

[1] q_e is the adsorbed amount of BG at the equilibrium (mg/g); C_e represents the equilibrium concentration of BG in solution (mg/L); Chi-square statistic test (χ^2) is calculated based on equation S1; q_m and q_s are the theoretical Langmuir and Sips maximum adsorption capacities (mg/g); K_L, K_F, K_S, K_T and K_D are constants corresponding to each isotherm model; R_L is the separation factor calculated according to Equation S2; n_F is the surface heterogeneity factor; c is Sips isotherm exponent; B_T is related to heat of adsorption (J/mol); E_S is the mean free energy (kJ/mol) and r^2 is the correlation coefficient(mol²/kJ²) [40,46].

[2] The decrease of $1/n_S$ values from 0.84 (at 300 K) to 0.56 (at 330 K) suggests that the increase of temperature might enhance the surface heterogeneity [40].

The dimensionless separation factor R_L (equilibrium parameter) is calculated based on Langmuir model constant (K_L), and was represented as the average of the calculated values for each initial dye concentrations (from 10 to 500 mg/L):

$$R_L = \frac{1}{1+K_L C_0} \,, \qquad \textbf{(S2)}$$

where K_L is the Langmuir adsorption constant and C_0 represents the initial dye concentration. The R_L parameter value provides information about the nature of the adsorption process ($R_L = 0$ indicates an irreversible adsorption, R_L ranging between 0 and 1 suggests a favorable absorption and $R_L = 1$ denotes a linear unfavorable absorption) [46].

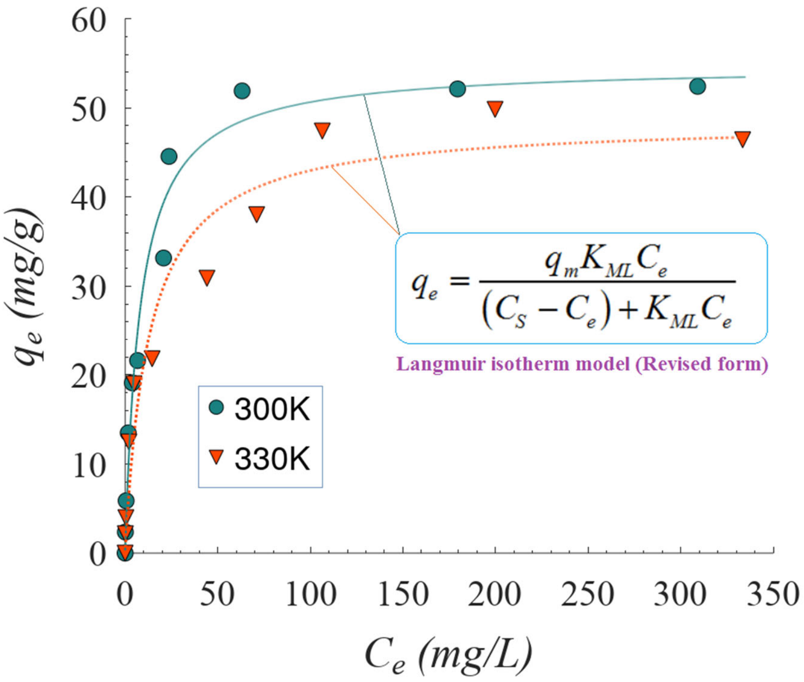

Figure S2. Adsorption isotherms data fitted to the revised Langmuir isotherm model (Azizian approach [36]), showing the adsorption equilibrium established between the surface of pistachio shells adsorbent and Brilliant green (BG) cationic dye; water solubility of BG dye is $C_S = 1\times10^5$ mg/L.

Table S5. Parameters of the revised Langmuir isotherm model determined for BG dye adsorption onto PS_{NaOH} biosorbent ($C_S = 1 \times 10^5$ mg/L (solubility for BG dye)).

**Temperature, T (K)**	q_m **(mg/g)**	K_{ML} **(dimensionless)**	χ^2**-test (error function)**

300K	54.743	1.2362×10^4	8.534
330K	48.491	7.7868×10^3	20.701

Note: C_S=1×10^5 mg/L or 0.2072 mol/L (solubility for BG dye)

Table S6. Analysis of variance (ANOVA) for the multiple regression model $\hat{Y}\left(x_1, x_2\right)$.

Source	*DF* [(a)]	*SS* [(b)]	*MS* [(c)]	*F-value* [(d)]	*P-value* [(e)]	R^2 [(f)]	$R_{adj}{}^2$ [(g)]
Model	5	7649.13	1529.83	26.51	0.0013	0.963	0.927
Residual	5	288.58	57.72				
Total	10	7937.71					

[(a)] degree of freedom; [(b)] sum of squares; [(c)] mean square; [(d)] ratio between mean squares;

[(e)] probability of randomness; [(f)] coefficient of determination; [(g)] adjusted coefficient of determination;

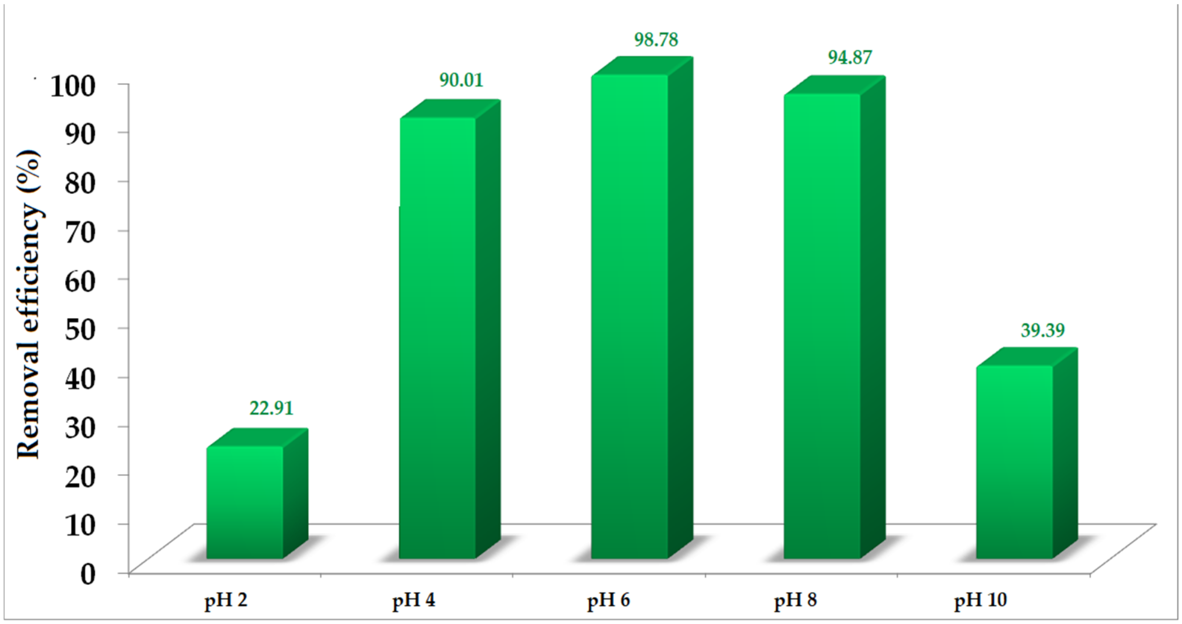

Figure S3. Effect of pH: removal efficiency of Brilliant Green (BG) from aqueous solutions of pH 2 and 4 (adjusted with 0.1M H_2SO_4) and pH 8 and 10 (adjusted with 0.1M NaOH) compared to naturally pH 6; optimal experimental conditions: SD = 4 g/L, C_0 = 10 mg/L, V = 50 mL, t =240 min).

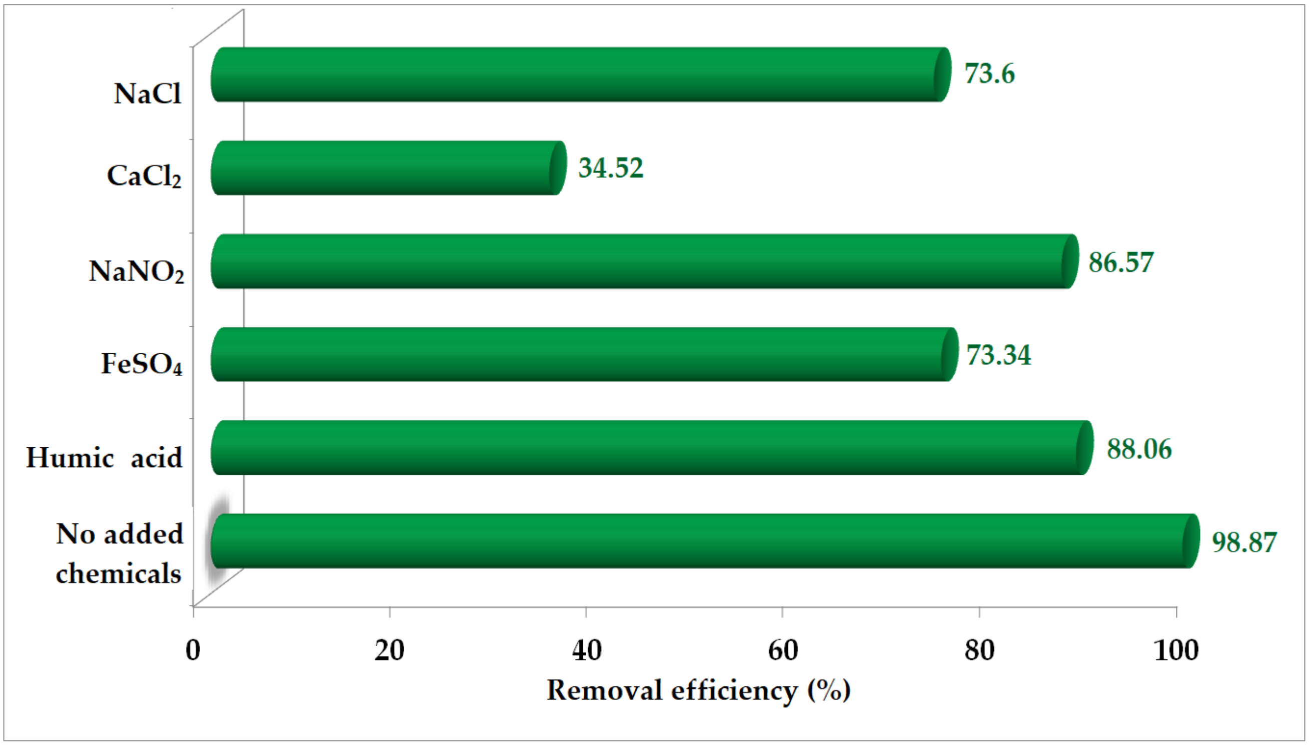

Figure S4. Removal efficiency of Brilliant Green (BG) from aqueous solutions in the presence of different salts (200 mg/L NaCl, $CaCl_2$, $NaNO_2$, or $FeSO_4$), and in the presence of organic matter (20 mg/L humic acid); optimal experimental conditions: SD = 4 g/L, C_0 = 10 mg/L, V = 50 mL, t =240 min.

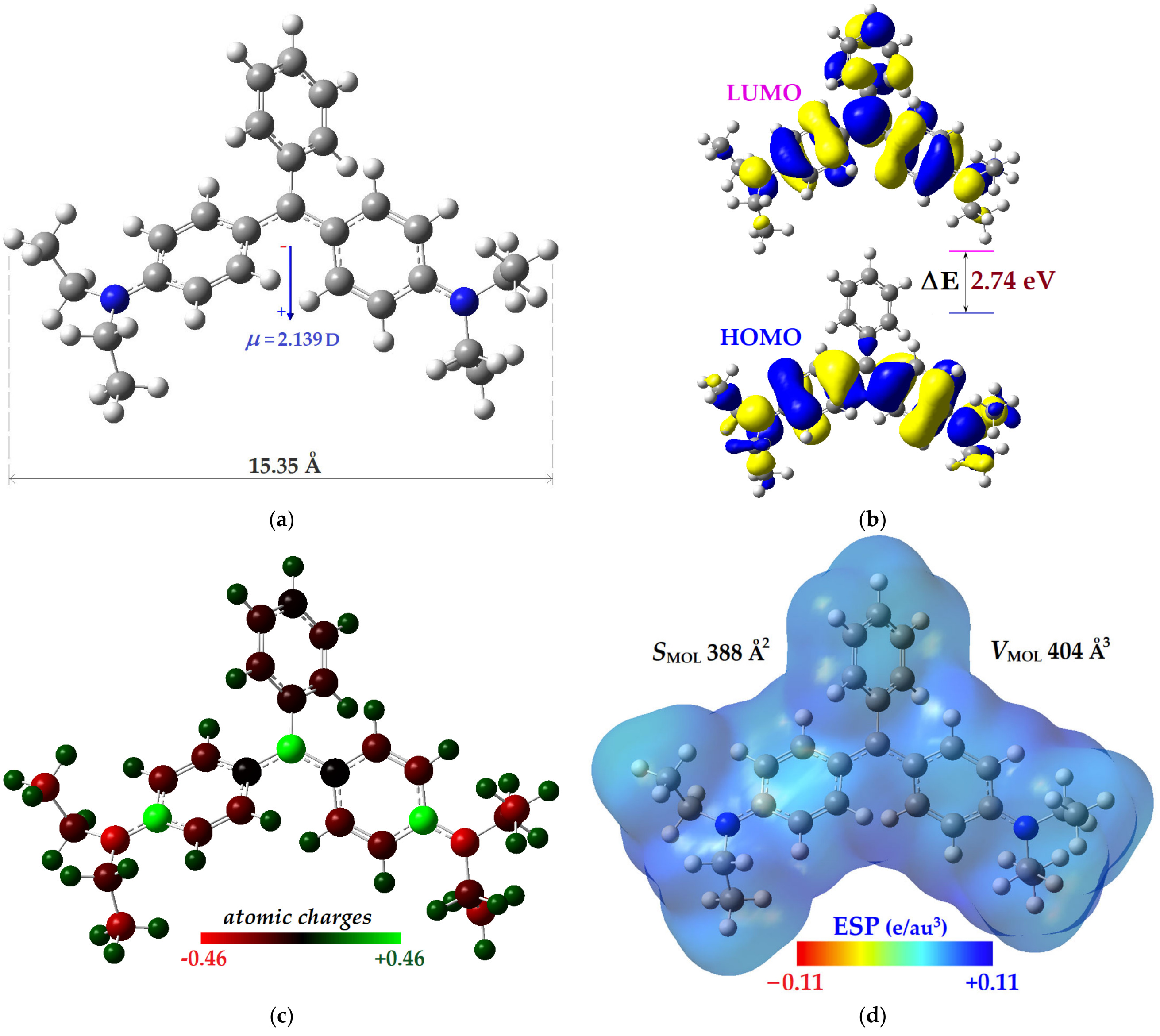

Figure S5. Molecular structure and particularities of BG molecule estimated computationally at DFT/APFD/6-311G(2d,p) level of theory for ground state S_0 (charge = +1, singlet): (**a**) optimized geometry and dipole moment; (**b**) frontier molecular orbitals (HOMO, LUMO); (**c**) partial atomic charges (Mulliken) distribution; (**d**) electrostatic potential (ESP) map.

The results of DFT computations comprise: the optimal geometry and dipole moment orientation for the BG molecule (Figure S5a), the patterns of the frontier molecular orbitals HOMO, LUMO (Figure S5b); the distribution of the partial atomic charges (Figure S5c); and the electrostatic potential (ESP) map in the vicinity of the molecular surface of BG (Figure S5d). Computational results unveiled the following features for the BG molecule, size of 15.35 Å and a dipole moment equal to 2.139 Debye (Figure S5a). The lobes of molecular orbitals (HOMO and LUMO) are delocalized onto aromatic rings, indicating a π-conjugated system. Hence, the configuration HOMO→LUMO was associated to π→π* electronic transition, revealing an energy gap equal to 2.74 eV (Figure S5b). Partial atomic charges (Mulliken) for the BG molecule are given in Figure S5c and rendered as color gradient. The estimated electrostatic potential (ESP) surrounding the BG molecule (cationic form) is depicted in Figure S5d. Thus, BG cationic dye with a molecular surface equal to 388 Å^2 (and a molecular volume of 404 Å^3) carries a positive electrostatic potential (Figure S5d). Additional molecular and reactivity descriptors for BG molecule (cationic form) are summarized in Table S7.

Table S7. Summary of molecular and global reactivity descriptors for BG molecule (cationic form); descriptors computed by DFT method at APFD/6-311G(2d,p) level of theory.

Molecular / Reactivity Descriptor	BG molecule (cationic form)
Empirical formula	$C_{27}H_{33}N_2$
System description	62 atoms, 208 electrons, +1 charge, singlet
Molecular mass (amu)	385.26
LUMO energy, ε_{LUMO}, (eV)	-5.564
HOMO energy, ε_{HOMO}, (eV)	-8.301
Energy gap: $\Delta E = \varepsilon_{LUMO} - \varepsilon_{HOMO}$, (eV)	2.737
Ionization potential: $I \approx -\varepsilon_{HOMO}$, (eV)	8.301
Electron affinity: $A \approx -\varepsilon_{LUMO}$, (eV)	5.564
Electronegativity: $\chi = ½ (I + A)$, (eV)	6.933
Chemical potential: $\mu = -\chi$, (eV)	-6.933
Chemical hardness: $\eta = ½ (I - A)$, (eV)	1.369
Chemical softness: $S = 1/\eta$, (eV^{-1})	0.731
Electrophilicity index: $\omega = \mu^2/2\eta$, (eV)	17.557

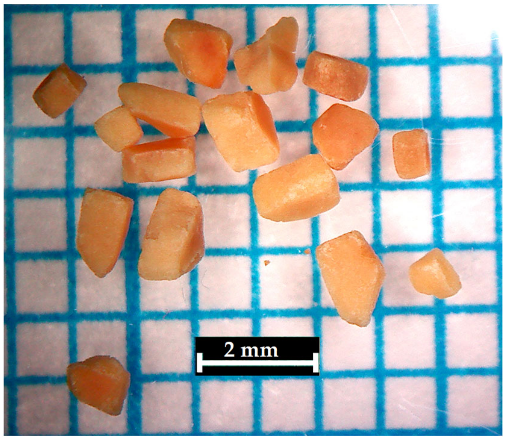

Figure S6. PS_{NaOH} grains image obtained by optical microscope (Conrad USB, Wels, Austria).
